# Liver-ing in your head rent free: peripheral ApoE4 drives CNS pathology

**DOI:** 10.1186/s13024-022-00569-1

**Published:** 2022-10-04

**Authors:** Lesley R. Golden, Lance A. Johnson

**Affiliations:** 1 Department of Physiology, University of Kentucky, Lexington, Kentucky USA; 2 Sanders Brown Center On Aging, University of Kentucky, Lexington, Kentucky USA

**Keywords:** Peripheral ApoE, ApoE4, Alzheimer’s disease, Cerebrovascular impairment, Blood–brain barrier, Liver

## Main text

The ε4 allele of Apolipoprotein E (*APOE*), which is carried by approximately one out of six individuals, remains the strongest known genetic risk factor for late-onset Alzheimer’s Disease (AD). Despite established roles in numerous peripheral disorders [1], the AD field has almost exclusively focused its *APOE* attention above the neck. This makes sense taken at face value, as the two major pools of ApoE protein – peripheral (hepatocyte-derived) and CNS (primarily astrocyte-derived) – appear to remain separated by the blood–brain barrier (BBB) [2, 3]. However, mounting evidence suggests that regardless of any physical separation between these two pools, peripheral ApoE may still notably impact CNS function [4, 5]. In the latest issue of *Nature Neuroscience*, Liu et al. further explore the effects of peripheral to CNS ApoE cross-talk, reporting detrimental outcomes on brain function and AD-associated pathologies following peripheral-only expression of ApoE4 [6].

The relative ease of accessing the blood or liver compared to the brain, together with the growing interest in ApoE as a therapeutic target, increases the need to understand if and how peripheral ApoE affects CNS function [7]. A handful of studies have begun to explore these questions, including a 2019 report where deletion of human *APOE* in hepatocytes did not affect cerebral amyloid pathology[8]. A separate study recently used mice transplanted with primary human hepatocytes to show that plasma ApoE4 levels were negatively associated with markers of synaptic integrity, neuroinflammation and insulin signaling [5].

In this recent manuscript, Liu et al. also step up to the challenge by employing a mouse model capable of liver-specific expression of human ApoE3 (E3) or ApoE4 (E4). These mice were bred onto an *Apoe* knockout background in order to isolate the effects of peripheral E3 and E4 on CNS function. In comparison to E3, E4 has been previously linked to cerebrovascular dysfunction and BBB breakdown, cognitive impairment, and gliosis [9–11]. Interestingly, Liu et al. show similar results when comparing the effects of *only* peripheral E3 vs. *only* peripheral E4 on CNS function. They demonstrate that mice expressing only liver-derived E4 had significant memory deficits, increased BBB leakage, cerebrovascular impairments, and increased vessel-associated gliosis when compared to mice expressing only liver-derived E3.

Using single-cell RNA sequencing, Liu and colleagues provide some insight into the potential mechanism(s) by which hepatic-derived ApoE could be affecting CNS function and BBB integrity. E4 is known to drive a host of cerebral transcriptomic changes, namely in genes related to neuroinflammation, carbohydrate and lipid metabolism, and cerebrovascular integrity [12–14]. Similarly, in this liver-only ApoE system, correlation analyses showed a strong link between E3 and genes involved with cell adhesion, synaptic connections, learning and memory, and carbohydrate metabolism. In contrast, E4 was associated with vasodilation regulation, lipid metabolism, cell survival, and energy homeostasis. Most notably, genes important for endothelial cell function, BBB integrity, and extracellular matrix (ECM) were downregulated in mice expressing liver-derived E4. In vascular cell-enriched populations, peripheral expression of E4 also reduced gene expression of pathways related to perivascular astrocyte end feet formation, increased inflammation in endothelial cells, and intensified gliosis. Altogether, the authors’ data suggest that the peripheral pool of E4 alone is sufficient to drive dysfunction in these pathways, thereby compromising BBB integrity and cerebrovascular function.

Alongside these transcriptomic changes, Liu et al. show alterations in the plasma proteome based on peripheral ApoE isoform expression. Hepatic-derived E3 was associated with modules involved in downregulation of proteasomal protein catabolic processes and lipid metabolism, and upregulation of cell–cell adhesion, fibrinogen complex, and protein secretion regulatory processes. The authors proposed that increases in Timp3, an ECM-binding protein upregulated in one of their modules, could mechanistically contribute to E3’s improved vascular and brain function. Hepatic expression of E4 on the other hand was significantly associated with downregulation of proteins involved in lipoprotein metabolism and lipid transport/clearance, while proteins involved in the immune response, complement activation, and fatty acid metabolism were upregulated in E4-enriched modules.

To elucidate the effects of peripheral ApoE on AD pathology, Liu and colleagues crossed their liver specific knock-in mice to amyloid-overexpressing APP mice. Excitingly, hepatic expression of E3 resulted in a decrease in amyloid plaque load compared to whole-body *Apoe* knockout controls. Conversely, expression of liver-derived E4 worsened amyloid pathology. Additionally, Liu et al. report reduced levels of synaptic density marker PSD-95 with expression of peripheral E4, but increased levels with expression of peripheral E3. The group validated their findings using an additional model of amyloidosis, namely 5xFAD mice transduced with AAV-albumin-ApoE3 or AAV-albumin-ApoE4, to determine how peripheral ApoE isoforms affect amyloid pathology in the presence of brain ApoE. Overall, their experiments suggest that cerebral amyloid load is affected by peripheral ApoE in an isoform-dependent manner (with E3 improving and E4 exacerbating pathology).

The concept of using young plasma to counteract age-related cognitive decline has shown promising results as a potential therapeutic strategy [15]. Liu et al.’s study expands upon this idea, incorporating the role of different ApoE isoforms and how they affect the therapeutic potential of using young plasma to target AD-pathologies. By intravenously injecting young E3 or young E4 plasma into aged, wild-type mice, they show that E4 impairs, while E3 improves, measures of associative memory, BBB leakage, and vessel-associated gliosis. Using iPSC derived endothelial cells (iBMECs), the authors recapitulate their findings by focusing on changes in endothelial cell function upon treatment with young E3 or E4 plasma. Through measures of trans-endothelial transport and bulk-RNA sequencing, they report E3-associated increases in barrier integrity compared with E4, along with E4-associated cerebrovascular impairment and dysregulation of genes involved with ECM and cell mobility pathways. However, co-treatment with young E4 plasma and Timp3 (the previously mentioned E3-associated ECM protein) together improved the weakened integrity of the iBMEC monolayer.

**Fig. 1:**
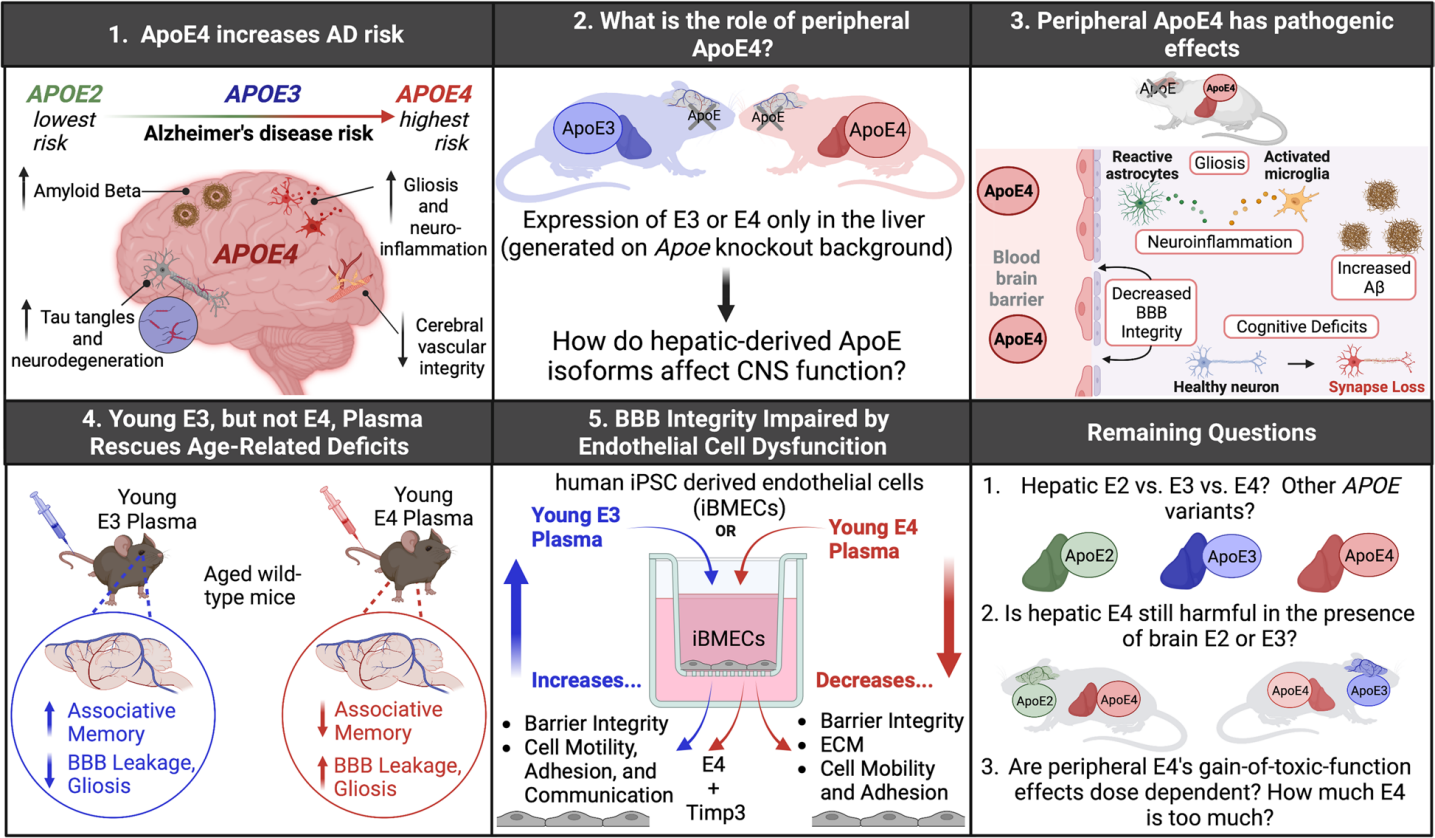
Apolipoprotein E4 (*APOE4*) increases Alzheimer’s disease risk and worsens pathology. However, the influence of peripheral ApoE isoforms on CNS function is still unclear. In their recent publication, Liu et al. leverage a novel mouse model expressing hepatic ApoE3 or ApoE4 on an *Apoe* knockout background to show that liver-derived E4, but not E3, has multiple pathogenic effects. Several of these effects, namely cognitive and cerebrovascular function, can be improved by treating aged mice with young E3 plasma, while E4 plasma exacerbates deficits. Additionally, young E3 plasma, or co-treatment with young E4 plasma plus Timp3, improved endothelial cell function in a human iPSC-derived endothelial cell system. This exciting study highlights the important interaction between peripheral ApoE and vascular-mediated AD pathogenesis, and raises several important additional questions for the field

In summary, Liu et al.’s recent paper highlights the role of liver-derived E4 in brain function, emphasizing an important interaction between peripheral ApoE and the neurovascular unit in AD pathogenesis. The authors’ discoveries raise several interesting questions and open new avenues to advance AD therapeutics (Fig. 1). For example, would hepatic expression of other *APOE* variants (such as *APOE2* or the putatively neuroprotective Christchurch or Jacksonville variants) be additionally protective against CNS pathologies? Could they do so in the presence of endogenous E4 (i.e. would injections of plasma with these protective *APOE* variants serve as an effective method to target and mitigate AD-pathologies)? Additionally, it would be interesting to establish if E4’s gain-of-toxic function effects are dose dependent, and if hepatic E4’s effects are equally as harmful in the presence of brain E2 or E3 as they were in *Apoe* deficient model used in the current study. While these and other important questions remain, the findings from this exciting paper solidify the idea that the effects of *both* CNS and peripheral pools of ApoE need be considered when designing therapies – particularly because liver-derived ApoE could be an accessible and effective target to mitigate AD-associated pathologies.

## Data Availability

Not applicable.

## Abbreviations

ApoE: Apolipoprotein E
AD: Alzheimer’s disease
BBB: Blood brain barrier
CNS: Central nervous system
E3: ApoE3
E4: ApoE4
ECM: Extracellular Matrix
